# Remote, autonomous real-time monitoring of environmental DNA from commercial fish

**DOI:** 10.1038/s41598-020-70206-8

**Published:** 2020-08-06

**Authors:** Brian K. Hansen, Magnus W. Jacobsen, Anne Lise Middelboe, Christina M. Preston, Roman Marin, Dorte Bekkevold, Steen W. Knudsen, Peter R. Møller, Einar E. Nielsen

**Affiliations:** 1 Section for Marine Living Resources, National Institute of Aquatic Resources, Technical University of Denmark, Vejlsøvej 39, 8600 Silkeborg, Denmark; 2 DHI, Agern Allé 5, 2970 Hørsholm, Denmark; 3 Monterey Bay Aquarium Research Institute, 7700 Sandholdt Rd, Moss Landing, CA USA; 4 NIVA-Denmark, Njalsgade 76, 2300 Copenhagen S, Denmark; 5 Natural History Museum of Denmark, University of Copenhagen, Universitetsparken 15, 2100 Copenhagen Ø, Denmark

**Keywords:** Biological techniques, Genetics, Molecular biology

## Abstract

Environmental DNA (eDNA) is increasingly used for monitoring marine organisms; however, offshore sampling and time lag from sampling to results remain problematic. In order to overcome these challenges a robotic sampler, a 2nd generation Environmental Sample Processor (ESP), was tested for autonomous analysis of eDNA from four commercial fish species in a 4.5 million liter mesocosm. The ESP enabled in situ analysis, consisting of water collection, filtration, DNA extraction and qPCR analysis, which allowed for real-time remote reporting and archival sample collection, consisting of water collection, filtration and chemical preservation followed by post-deployment laboratory analysis. The results demonstrate that the 2G ESP was able to consistently detect and quantify target molecules from the most abundant species (Atlantic mackerel) both in real-time and from the archived samples. In contrast, detection of low abundant species was challenged by both biological and technical aspects coupled to the ecology of eDNA and the 2G ESP instrumentation. Comparison of the in situ analysis and archival samples demonstrated variance, which potentially was linked to diel migration patterns of the Atlantic mackerel. The study demonstrates strong potential for remote autonomous in situ monitoring which open new possibilities for the field of eDNA and marine monitoring.

## Introduction

Analysis of aquatic environmental DNA (eDNA) has gained momentum in recent years due to the method´s potential for rapid and cost-effective biomonitoring circumventing collection and visual identification of living specimens^1–3^. This has led to much research over the past decade in relation to detection and quantification of aquatic macro-organisms in both freshwater^2,4,5^ and more recently in marine environments^1,3,6^. Despite the apparent benefits, eDNA surveys still require manual sampling of water at designated sampling sites, which constrains eDNA based marine monitoring in remote and offshore areas due to the associated boat-costs^7,8^. Such limitations also prevent the acquisition of standardized eDNA time series on a scale from days to months, potentially leading to failure to detect important temporal and spatial changes in species occurrence and abundance, which is of particular relevance for rare and migratory species^9^. Moreover, there is presently a considerable time lag in eDNA from field sampling to the result is available which delays and hampers management efforts. Despite the apparent need for time series and real-time data to accurately monitor and manage larger organisms in the oceans, completely autonomous in situ sampling, analysis and remote reporting of eDNA has not been conducted so far^10^.

One field of marine monitoring where the potential of eDNA has been highlighted as a valuable supplement or even replacement for existing monitoring methods is in relation to marine fisheries^3,11^. Here, extensive monitoring is conducted worldwide in order to describe spatial and temporal dynamics of fish stocks, which is key for sustainable resource management. Still, gaining sufficient information through traditional monitoring of living aquatic organisms remains expensive and challenging^7^. Recent advances in oceanographic instrumentation has made remote and automated collection of advanced marine data possible^12^. These include “ecogenomic sensors^13^” which allow in situ genetic analysis^14,15^. One such instrument is the 2nd generation Environmental Sample Processor (ESP), which is a DNA laboratory that can be deployed subsurface for several months facilitating automated collection, filtration, extraction and DNA analysis of ambient water samples. In addition, two-way communication secures remote control of the instrument and allows results to be available on land few hours after initiation of sampling^14,15^. Here we report and evaluate the performance and sensitivity of a modified ESP for autonomous collection, detection and quantification of eDNA particles from four commercially important fish species in a large marine mesocosm. Specifically, the primary aim was to evaluate the ability of the ESP to perform real-time eDNA quantification, and assess the consistency of these estimates in a highly abundant species. The secondary aim was to evaluate the sensitivity of the ESP for detection of species with very low abundance, i.e. “rare species”.

**Figure 1:**
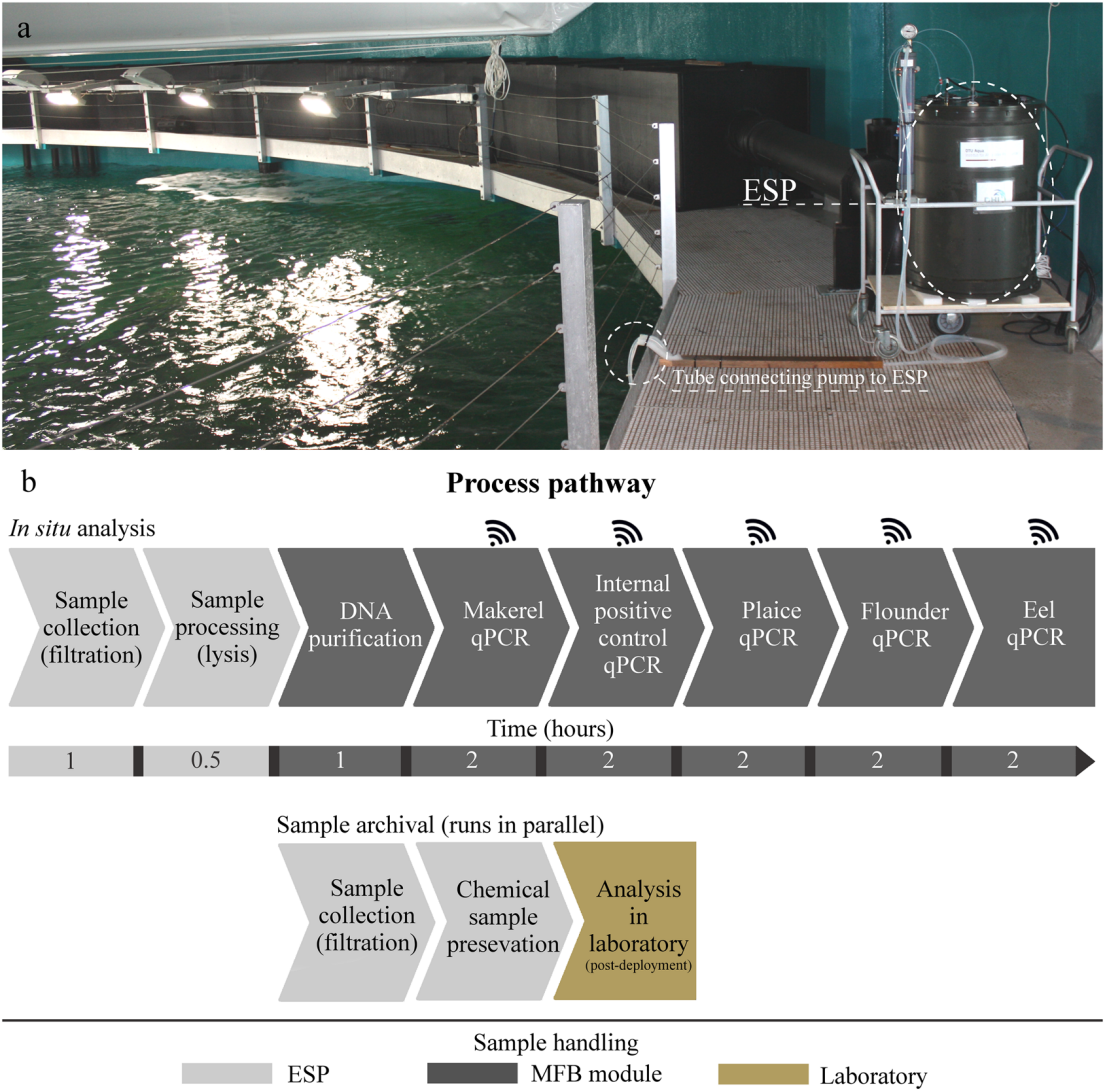
ESP experimental setup and process pathway. (**a**) ESP setup at the mesocosm (see methods for details). (**b**) Schematic illustration of the sampling and analytical processing during deployment of the ESP. The duration of each process is an estimate and illustrates the analysis of a 1.5 L sample.

To test this, a controlled experiment was conducted in a 4.5 million liter mesocosm aquarium tank containing 27 fish species native to the Northeast Atlantic (Supplementary Table S1). The ESP was placed adjacent to the mesocosm, and an external pump was used to deliver surface water to the ESP (Fig. 1a). The ESP analyzed eDNA from four common Northeast Atlantic fish species, Atlantic mackerel (*Scromber scrombus*), European plaice (*Pleuronectes platessa*), European flounder (*Platichthys flesus*) and European eel (*Anguilla anguilla*), hereafter referred to as mackerel, plaice, flounder and eel respectively. During the 51-day deployment, the ESP conducted 30 sampling events. Each event was composed of one complete in situ analysis, consisting of water collection, filtration, DNA extraction and qPCR analysis, followed by one archival sample collection, i.e. water collection and filtration followed by chemical preservation and onboard storage (Fig. 1b). The archival sample acquisition started when sample collection and processing of the sample for the in situ qPCR analysis was completed, thus running in parallel with the in situ qPCR analysis. From day 42 to day 49, an extra archival sample was collected each day with water sampling in the evening (starting at 20:00). Laboratory based qPCR results from morning and evening archival samples are referred to as Archival-M and Archival-E, respectively, while the in situ qPCR analysis from the ESP is referred to as in situ analysis (see complete sampling scheme in Supplementary Table S2). During deployment, each in situ analyses sequentially ran each target species and an internal positive control (IPC) to test for reaction inhibition (Fig. 1b).

## Results and discussion

**Figure 2:**
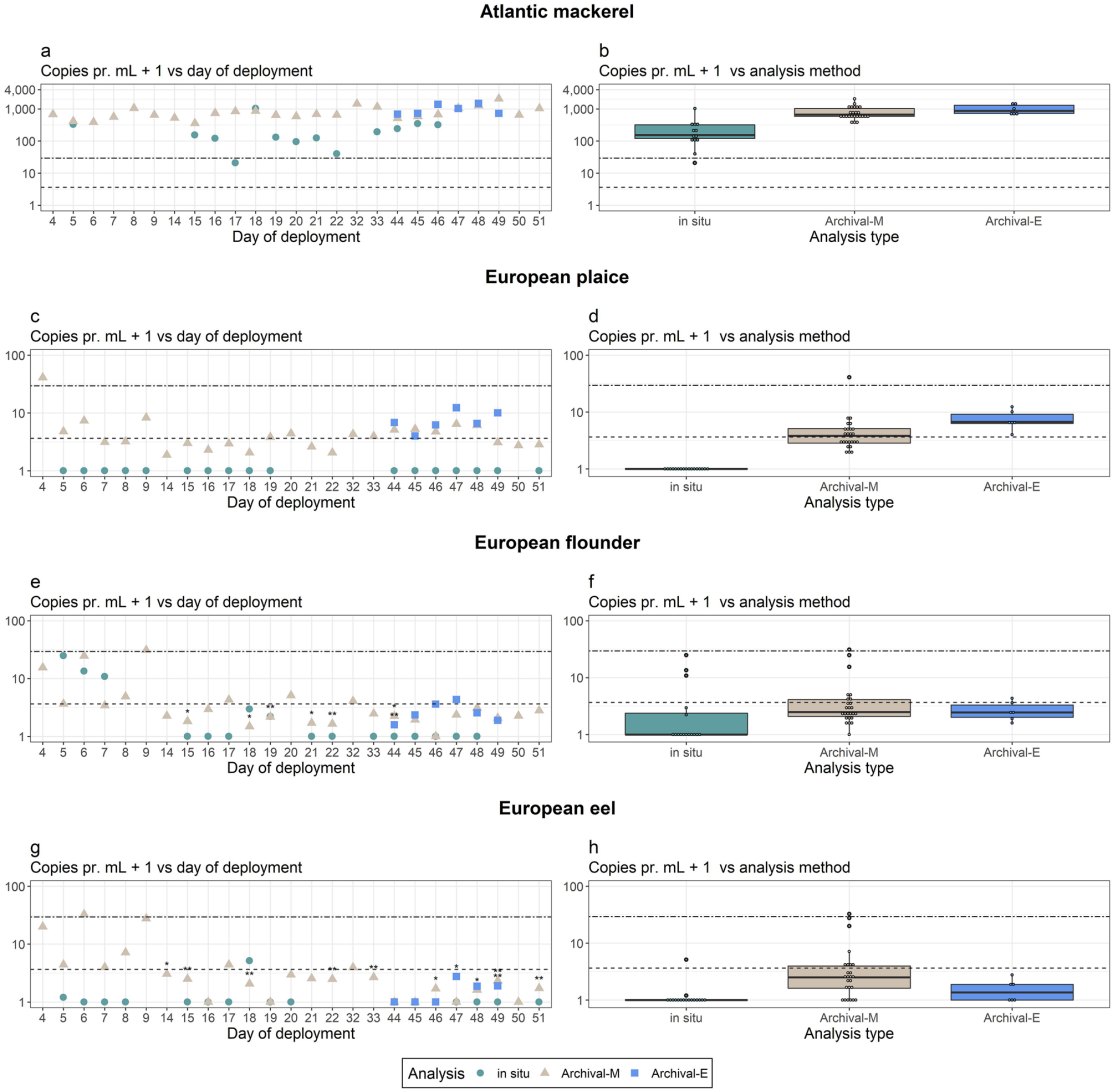
Time series eDNA results for Atlantic mackerel (**a**,**b**), European plaice (**c**,**d**), European flounder (**e**,**f**) and European eel (**g**,**h**). (**a**,**c**,**e**,**g**) Time series data for the 51-day deployment. (**b**,**d**,**f**,**h**) Boxplots of results during deployment, each dot represent a sample . For both plots the dashed line (− −) illustrate the LOQ for Archival-M and Archival-E and the two typed dashed line (∙ −) is LOQ for the in situ analysis. (*) are blank reactions (of three) for each sample.

Mackerel, which was by far the most abundant of the target species in the mesocosm (Supplementary Table S1), was consistently detected in quantifiable amounts (above limit of quantification, LOQ) from both the in situ analysis and at the post-deployment qPCR analysis of archived samples in the laboratory (Fig. 2a). The LOQ being the lowest concentration of positive controls that all amplify for the qPCR setup^16^. The in situ analysis of mackerel DNA ranged from 1,036 copies/mL to unquantifiable detection (one sample) with an average concentration across the complete sampling period of 260 copies/mL (SD ± 264). Archival samples ranged from 2073 copies/mL to 355 copies/mL with an average of 843 copies/mL (SD ± 382). The observed variability among samples is expected to originate from the natural eDNA distribution variability as well as some technical variability in the in situ analysis. For the in situ analysis, there is a tradeoff between showing reaction variability and testing for multiple species. Additionally, copy number estimates were significantly different between the in situ analysis and the archived samples (Fig. 2b; general linear model, *P*_Archival-M_ = 1.6·10^−8^, *P*_Archival-E_ = 1.2·10^−6^, df = 41), while estimates for Archival-E and Archival-M did not differ significantly (Fig. 2b; general linear model, *P* = 0.16, df = 29). The difference between in situ analysis and the archived samples was not due to a lower extraction efficiency of the MFB module compared to the benchtop extraction. Instead, the lower in situ values may be due to software or hardware differences in calculating cycle thresholds between the MFB and the laboratory based qPCR analysis, which impact qPCR standards and copy number estimates^17,18^. Alternatively, the difference could also be linked to the behavior of mackerel, which displayed strong semidiurnal migrations between the bottom and surface of the mesocosm, triggered by an artificial sunrise at 09:00 each day. Water sampling of the in situ analysis was conducted prior to diel migration and archival sampling was conducted after. Small scale spatial variations has often been reported in eDNA studies^1,19,20^, but not specifically linked to semidiurnal behavior.

The low abundance, “rare”, species were rarely (eel, N = 2) or never (plaice and flounder) detected with the in situ analysis. However, for the archival samples, plaice, flounder and eel were detected in 100%, 97% and 71% of the samples (Fig. 2c–h). There was no clear tendency for plaice to have higher DNA abundance despite having higher abundance than flounder and eel. Likewise, introducing a single eel to the tank did not result in consistent changes in detection of eel eDNA. A number of factors have to be considered to explain the eDNA results for the “rare” species. Besides their low abundance, they are benthic, and thereby occur with the furthest distance to the pump intake. Additionally, the rare species were analyzed last in the process pathway (Fig. 1b). This leaves up to 11 h for potential loss of the target due to DNA degradation. Thus, the higher copy number in archived samples likely relate to better DNA preservation via the addition of RNA*later* immediately following sampling. Hence, for future deployments it could be advisable to prioritize rare target species first in the analytical sequence. The sporadic findings of eel DNA even in periods where eel should not be present in the tank is likely due to exogenous input from constant replenishment of mesocosm water during the experiment. An exogenous DNA source may also be relevant for DNA signals of the two other rare species in our experiment, plaice and flounder, and highlights the general challenge in eDNA analysis of determining the source of the detected DNA in aquatic systems^20–22^. Here, at least, we are able to assess and quantify the potential bias (see Supplementary Fig. S2).

To our knowledge, this is the first proof of concept utilizing an “ecogenomic sensor” for remote, automated eDNA sampling and in situ analysis to provide time series of eDNA presence and abundance in close to real-time for marine macro-organisms. The 51-day autonomous deployment here illustrates that the approach is technically sound, feasible and robust. Still, there is room for improvement of the methodology, which will benefit from further optimization in particular regarding preservation of the DNA during in situ analysis leading to an overall improvement of the ability to detect and quantify DNA from rare species. This could for example be accomplished by utilizing an elution buffer, which preserves DNA better than water, through increasing sampling volume or by PCR multiplexing so the “queueing time” from extraction to PCR becomes shorter (Fig. 1b). Thus, initial practical application in the ocean may mainly be feasible for targeting relatively common species to document shifting distributions and abundance. In addition, analysis of eDNA from the archival samples has demonstrated the very large potential of the ESP for long term, continuous and cheap sampling of aquatic eDNA from remote and inaccessible marine areas. Such samples can subsequently be used to complement in situ qPCR results, but also serve as a source of DNA for new studies targeting a wide range of marine organisms from virus to whale. The insights gained from archival sampling has unpreceded perspectives for offshore marine monitoring and could revolutionize monitoring of spatial and temporal dynamics of entire marine ecosystems. Moreover, the ESP technology can relatively easily be combined with other environmental sensors measuring physical, chemical or biological parameters, which can provide unprecedented resolution for an integrated understanding of the ecology of eDNA and improve biological inferences. We expect the application of eDNA coupled with the well-tested ESP technology will provide a wealth of new opportunities for future remote ocean monitoring, potentially providing real-time insights into the relationships between short and long term changes in the environment and the distribution of marine macro-organisms, which today remain unexplored due to costs of offshore sampling.

## Methods

### Mesocosm

The Oceanarium tank at the North Sea Oceanarium (Hirtshals, Denmark) contained 27 different fish species including bony fishes, rays and sharks. The number of individuals of the four species targeted by the ESP was highly varied with approximately 1,400 (mackerel), 50 (plaice), 1 (flounder) and 0–1 (eel). During the deployment of the ESP a live European eel (~ 200 g) in a fyke net was added to the mesocosm from day 20 to 25 and from day 44 to end of deployment. Intake water and fish feed were identified as potential vectors of exogenous DNA introduction. Approximately 10% (~ 450,000 L/day) of the mesocosm volume is replaced each day with water from the nearby ocean. Intake water is mechanically filtered through sand, but with no chemical or UV treatment that removes DNA, thus potentially introducing exogenous DNA from fish occurring near the ocean intake. To test for this, intake water (3 × 1 L) was collected directly from the inlet pipe to the mesocosm (day 20). Using sterile single use 60 mL syringes (fisher scientific, USA) water was pushed through Swinnex filter holders (SX0002500, Millipore, USA) fitted with a 0.22 µm duropore filter (GVWP02500, Millipore, USA). After collection (~ 1 h) filters were stored on ice for 2 h in a freezing bag and hereafter stored at − 20 °C. DNA extraction and analysis was conducted as described below for the archival samples. Fish in the Oceanarium tank were fed in excess of 69 kg/week of 2 mm dried pellets and maximum 1 kg/week of 6 mm pellets. Samples of food pellets were crushed, weighed (16.1–25.9 mg/extraction) and DNA extracted using a DNeasy Blood & Tissue Kit (Qiagen, USA) according to the manufacturer’s suggestions, with a final elution volume of 160 µL. Samples were diluted (1:100) with nuclease free water prior to qPCR analysis (described below). Potential target DNA concentrations in the mesocosm originating from feed were calculated using an exponential decay model using DNA degradation rates from Sassoubre and colleagues^21^, who studied similar species in a similar mesocosm environment (Supplementary Table S3):

$$ \frac{dN}{{dT}} = - \beta N\,{\text{with}}\,{\text{solution}}\,N\left( t \right) = N_{t} e^{ - \beta t} $$

where β is the decay constant and N is the number of DNA copies at time t. The water in the mesocosm was circulated through a trickling filter system with a velocity of 700 m^3^/h (turnover time of ~ 6.4 h) to facilitate decomposition of organic molecules (including DNA).

### ESP

The ESP is an electromechanical fluidic system that allows for autonomous water sample collection, sample processing and DNA analysis using qPCR^14,15^. The ESP was equipped with a microfluidics block (MFB)^15^ which contained a qPCR (quantitative PCR) module for detection and quantification of eDNA from the four target species. Filtration of a water sample is conducted with a titanium filter holder called a “puck”^15^, which contained a 0.22 µm filter. Water is repeatedly pulled through the filter and puck using a 25 mm syringe, until a user-defined volume is reached. Hereafter, the ESP can either (1) process and analyze the sample in situ or (2) archive it for later laboratory based analysis^15,23^. Each puck is only used once during deployment and after each sample is processed the system is decontaminated (see below). The ESP can hold up to 132 pucks. For each qPCR analysis, the ESP uses two pucks and for each archival samples, the ESP uses one puck. All fluidic movements within the instrument are controlled by syringes. The analytic data and all mechanical movements were logged and stored onboard as text files. While deployed, the ESP was set to automatically upload data when in situ analysis completed. All reagents, ESP components and methods were tested prior, during and after the mesocosm deployment of the ESP to ensure data integrity are detailed in the following sections.

### Assay validation

All assays were tested and optimized for both in situ analysis and laboratory based qPCR analysis, using a StepOnePlus Real-Time PCR System (Life Technologies, USA). Four species-specific qPCR assays^3^ for the target species (mackerel, plaice, flounder and eel) were applied except for a modification of the forward primer of the assay targeting mackerel to improve specificity. For all assays a double-quencher probe, 5′FAM/ZEN/3′IBFQ (Integrated DNA technologies, USA) was used to improve delta fluorescence (Supplementary Table S4). Assay specificity was tested with DNA from closely related and co-occurring species using 10 µL qPCR reactions, consisting of 1 µL DNA, 5 µL AccuPrime SuperMix I (Thermo Fisher Scientific, USA), 3 µL primer/probe solution and 1 µL nuclease free water (Supplementary Tables S4 and S5). DNA for non-target species was standardized to 1 ng/µL (1 ng DNA in each reaction) using a Qubit 2.0 fluorometer (Invitrogen, USA) with the dsDNA Broad Range kit (Invitrogen, USA). All laboratory based analyses including no template controls (NTC) were run in triplicates with the following thermocycling conditions: 95 °C for 1 min and 15 s, followed by 40 cycles of 95 °C for 15 s and 60 °C for 30 s. To assess cross instrument assay efficiency and sensitivity, synthesized double-stranded nucleotide amplicons, gBlocks (Integrated DNA technologies, USA) were used. gBlock copy number/µL was calculated from Qubit 2.0 fluorometer concentrations and subsequently diluted to make a standard qPCR curve. For each qPCR assay a batch of assay mix and a complimentary master-mix was produced and shared between the ESP and the laboratory analysis. Prior to the deployment a standard dilution series (6·10^5^–6·10^1^ copies/reaction) was run for each assay in the laboratory in 30 µL reactions to assess sterility and performance of the reagents. The lower limit of detection and qPCR efficiency for each target and platform is listed in supplementary Table S4.

### Deployment setup

During the deployment the ESP was placed 1 m above the water surface in the mesocosm from the 26th of January 2018 to 18th of March 2018 (see Fig. 1a). To minimize potential exogenous DNA from the fish feed, the ESP was set to sample water prior to feeding each day thereby maximizing the time between feeding and water sampling (20 h). However, time from feeding to sampling was only 7 h for evening archival samples (Archival-E). Water sampling and feeding were conducted at opposite sides of the mesocosm (~ 33 m). The ESP was originally designed for submerged water sampling, so an external sampling module, consisting of a phase separator and a pump (Typhoon DTW 50ft, Proactive, USA), was used to transfer water from the mesocosm to the ESP^14^. The pump, installed 2 m subsurface in the tank, transferred the water to the phase separator, which was pressurized by restricting outflow (~ 10 psi) and aided in ESP water filtration by creating a pressure head. After each sampling, the sample lines were flushed and the phase separator drained completely, hence minimizing cross-contamination. In addition, the external sampling module was automatically turned on by the ESP and self-flushed (~ 10 L/minute) for 5 min prior to initiation of sampling. Further, a copper screen was fitted to the ESP water intake to prevent large particles (> 1 mm) from entering the intake.

### In situ analysis

For each sample up to 1.5 L of seawater was filtered through a 0.22 µm duropore filter (GVWP02500, Millipore, USA) contained in a puck. Hereafter, 1.5 mL of a guanidine thiocyanate-based buffer^13^ was added to the filter with retained particles and heated to 85 °C for 5 min to lyse cellular material. The unpurified lysate was filtered through a second filter puck (containing a 0.22 μm durapore filter). To ensure sufficient and accurate prime only a portion (250 µL) of the unpurified lysate was mixed with 225  µL of SPE diluent (333 mM sodium acetate pH 5.2 in 200 proof ethanol). 400 µL of this mix was transferred to the MFB module for DNA purification using solid phase extraction. On the MFB module, to extract DNA, the lysate was passed through a custom made silica-packed HPLC column held at 55 °C and then subsequently rinsed with a column wash buffer (80 mM NaCl, 10 mM Tris–HCl pH 7.8, 504 mM EDTA, in 70% (v/v) 200 proof ethanol). After purification, the extracted DNA was eluted in 60 µL of nuclease free water (for details see Preston et al. 2011). The eluate was used in qPCR reactions to determine the presence of the four target species. All in situ analyses were carried out in 30 µL reaction volumes composed of 6 µL DNA, 18 µL enzyme mix (15 µL of AccuPrime SuperMix I (Invitrogen, USA) and 3 µL nuclease free water) and 6 µL primer–probe mix (Supplementary Table S4). For the in situ analysis, one reaction per sample was run for each species. The thermocycle conditions for the in situ analyses comprised an initial hold at 94 °C for 75 s, followed by 42 amplification cycles of annealing and extension at 60 °C for 30 s, and denaturation at 94 °C for 15 s. After each qPCR analysis the MFB cleaned itself with a 0.9% sodium hypochlorite solution and nuclease free water. Between each sample, both for in situ and archival samples, the entire fluidic pathway was cleaned using the same treatment. The ESP only collected the raw fluorescence data from the qPCR reactions, which was both stored and uploaded as a text file. Ct values and copies/mL was obtained from interpolating the fluorescence data with the pre and post-deployment standards.

Pre- and post-deployment qPCR standard curves were generated on the qPCR module on the MFB from dilution series of gBlocks to assess performance and stability of reagent across the deployment period. The pre-deployment standard was run from 6 × 10^5^ to 6 × 10^2^ copies/reaction in triplicates for all assays except the mackerel standard, which was run in duplicates. Post-deployment standards were run in duplicates and in some cases more times for the 6 × 10^2^ copies/reaction standard, which showed inconsistent results for plaice, flounder and eel. The average slope and intercept of the pre- and post-deployment standards were used to estimate copies per reaction (Supplementary Table S4). During the deployment several of the assay reagents lost prime and thus no data were reported (Supplementary Fig. S1a–h). While reagent issues were easily identified remotely, the ensuing procedure of repriming consumed additional reagent. To ensure sufficient reagent availability for samples, standards and negative controls were limited to duplicates for some assays. Prior to deployment, MFB DNA extraction efficiency was tested by sharing a sample between the MFB and a benchtop extraction kit (see section on DNA extraction from archival samples below for details) and measuring the DNA yield on a Nanodrop spectrophotometer (Thermo Fisher Scientific, USA). The MFB extraction provided a slightly higher extraction efficiency (45.95 ng/µL) then the benchtop extraction (38.23 ng/µL).

### Archival samples

Each sample collected for archiving was filtered through a puck containing a 0.22 µm duropore filter (GVWP02500, Millipore, USA) on top of a 0.45 µm (HAWP02500, Millipore, USA) filter. The purpose of the 0.45 µm filter was to ensure that the 0.22 µm remain soaked with RNAlater for appropriate DNA preservation. Immediately after filtration archival samples were evacuated of seawater and preserved with 4 mL of RNA*later* (Life Technologies, USA) for 2 min each, before RNA*later* was evacuated, and the preserved sample stored onboard the ESP at RT (17–18 °C). After instrument recovery, filters were transferred to a freezer and stored at − 20 °C until DNA extraction. Laboratory based DNA extraction and analysis mimicked those on the ESP, thus using a modified lysis and purification protocol using the DNeasy Blood and Tissue kit (Qiagen, USA) column. For DNA purification and final elution equivalent volumes to the ESP were used. For subsequent laboratory analyses of the archived DNA, all qPCR reactions were scaled down to 10 µL reactions to allow analysis in triplicates and analyzed on the StepOnePlus under thermocycling conditions mimicking the ESP. The triplicate 10 µL reaction setup utilized for the archival samples enables equal detection and quantification ability as the singlicate 30 µL in situ reaction setup by analyzing a total of 6 µL DNA in both procedures.

### Contamination and exogenous DNA

Prior to, during and after ESP deployment, quality assurance and control procedures were incorporated to ensure data integrity (Supplementary Information). Two tests were implemented to assess contamination. For the total in situ ESP processing pathway from sample collection to qPCR analysis, we conducted “core negative” (N = 7) controls, where the entire process is run without taking in water from the tank to assess potential internal contamination from previous samples. “Core negatives” were run pre-, post-deployment and at five additional times (day 3, 4, 14, 32 and 50). To assess potential contamination inside the MFB module^14^ (from DNA purification to qPCR analysis) three NTC’s were run with nuclease free water instead of column eluted DNA from filters. Prior to and after deployment, none of the core negatives amplified, while NTC amplification was observed on day 3. Data from day 1–3 was thus excluded from further analysis (see Supplementary Fig. S1). Mackerel assays showed signs of contamination on day 4, but amplification was below the levels from tank samples. After that, core negatives were completely negative on day 14 (Supplementary Fig. S1). For the flounder assays amplification was seen in the core negative on day 14, but not in the two flanking negative controls on day 4 or 32. Therefore, in situ detections from days 4–32 were excluded. Archived samples are expected to be relatively unaffected by contamination due to the processing pathway of the ESP (see section on ESP). Likewise, exogenous DNA from intake water, and fish feed used in the mesocosm was assessed. As expected, analysis of the intake water from the North Sea, showed traces of eDNA from all target species (Supplementary Fig. S2), albeit predominately estimated at below LOQ. qPCR analysis of feed pellets showed DNA from mackerel, plaice and flounder. Based on the DNA degradation model, the maximal contribution of exogenous DNA at the start of the sampling events were 27 to 67, 5 to 13 and 3 to 8 copies/ml for the respective species during day sampling and 99 to 137, 20 to 27 and 12 to 17 copies/ml respectively during evening sampling (Supplementary Table S3). However, visual observations strongly suggest that all pellets were consumed within seconds after introduction. A small proportion of the feed DNA is likely to escape complete degradation in the digestive tract of the fish and may be reintroduced to the water via feces, but in a much lower concentration^22^. Further, water filtration of the mesocosm (700 m^3^/hour—turnover time 6.4 h) mitigates buildup of organic particles (including feces). Although bacterial degradation of eDNA is not directly documented^24,25^, the mesocosm filter system is nonetheless still expected to remove particles containing eDNA that would otherwise have been suspended in the water.

### Statistical analysis and visualization

Data analysis and graphs was performed in R studio V. 1.1.456. Graphic representations of the data was made with the “ggplot2”, “scales”, “grid” and “gridExtra” packages. All illustrative and image editing work was conducted using GIMP version 2.8.18.

### Ethics statement

DTU has implemented the rules laid out in the EU-directive regarding animals in research (2010/63/EU – on the protection of animals used for scientific purposes). The general rule in Denmark is that only invasive procedures, like surgical implants, chemical stress or behavioral studies with inflicted pain require specific permission by the national Board for animals in research, thus a specific permission to use live fish for purposes like in the present study, cannot be sought. The experimental procedure with disturbance and handling of a live animal was conducted in accordance with local rules and regulations and fully comply with the DTU guidelines. Hence, no animal welfare or animal use permits were required for this study.

## Supplementary information

Supplementary information.
