# Supplementary material for: Remote, autonomous real-time monitoring of environmental DNA from commercial fish
Source: Sci Rep. 2020 Aug 6;10:13272. doi: 10.1038/s41598-020-70206-8 (PMC7413362; doi:10.1038/s41598-020-70206-8)
Supplement: Supplementary file 1 — Supplementary information. [file 41598_2020_70206_MOESM1_ESM.pdf]

Supporting information for the article

Remote, autonomous real-time monitoring of environmental DNA from commercial fish

Brian K. Hansen[1], Magnus W. Jacobsen[1], Anne Lise Middelboe[2], Christina M. Preston[3], Roman Marin III[3], Dorte Bekkevold[1], Steen W. Knudsen[4,5], Peter R. Møller[5] & Einar E. Nielsen [1]

[1]Section for Marine Living Resources, National Institute of Aquatic Resources, Technical University of Denmark, Vejlsøvej 39, 8600 Silkeborg, Denmark.

[2]DHI, Agern Allé 5, 2970 Hørsholm

[3]Monterey Bay Aquarium Research Institute, 7700 Sandholdt Rd, Moss Landing, California, United States of America

[4] NIVA-Denmark, Njalsgade 76, 2300 Copenhagen S

[5]Natural History Museum of Denmark, University of Copenhagen, Universitetsparken 15, 2100 Copenhagen Ø, Denmark

Corresponding author: bkha@aqua.dtu.dk

Running title: Autonomous *in situ* eDNA analysis

Supplementary table S1: Mesocosm species list

Common name	Scientific name	Abundance	Fish/m^3
**Teleost**			
Atlantic mackerel[a]	*Scomber scombrus*	1400	3.11E-01
Corkwing wrasse	*Symphodus melops*	150	3.33E-02
European seabass	*Dicentrarchus labrax*	55	1.22E-02
European plaice[a]	*Pleuronectes platessa*	50	1.11E-02
Garfish	*Belone belone*	50	1.11E-02
Greater weever	*Trachinus draco*	50	1.11E-02
Atlantic horse mackerel	*Trachurus trachurus*	30	6.67E-03
Atlantic bonito	*Sarda sarda*	23	5.11E-03
Whiting	*Merlangius merlangus*	20	4.44E-03
Ballan wrasse	*Labrus bergylta*	10	2.22E-03
Common sole	*Solea solea*	9	2.00E-03
Tub gurnard	*Chelidonichthys lucerna*	6	1.33E-03
Grey gurnard	*Eutrigla gurnardus*	6	1.33E-03
Gilthead seabream	*Sparus aurata*	4	8.89E-04
Lemon sole	*Microstomus kitt*	4	8.89E-04
Mullet	*Mugilidae spp.*	3	6.67E-04
Surmullet	*Mullus surmuletus*	1	2.22E-04
European flounder[a]	*Platichthys flesus*	1	2.22E-04
Ocean sunfish	*Mola mola*	1	2.22E-04
European eel[a]	*Anguilla Anguilla*	0-1	2.22E-04
**Elasmobranchs**			
Picked dogfish	*Squalus acanthias*	37	8.22E-03
Thornback ray	*Raja clavata*	15	3.33E-03
Lesser spotted dogfish	*Scyliorhinus caniculus*	12	2.67E-03
Nursehound	*Scyliorhinus stellaris*	8	1.78E-03
Tope shark	*Galeorhinus galeus*	6	1.33E-03
Starry smooth-hound	*Mustellus asterias*	4	8.89E-04
Smooth-hound	*Mustellus mustelus*	2	4.44E-04
Spotted ray	*Raja montagui*	2	4.44E-04

[a]Species targeted by eDNA analysis.

Supplementary table S2: Sampling scheme for total deployment

Sampling scheme for all collected water samples. Sample type: N refers to normal sampling, i.e. in situ analysis followed by collection of Archival-M sample. C refers to an in situ core negative analysis followed by Archival-M sampling. N + E refer to normal sampling combined with an Archival-E sample. Comments: a, Decrease in sampling power for the external pump. b, Malfunction of external pump. c, Replacement of external pump. d, These are volumes recorded by the ESP, but the actual samples are likely similar or smaller than recorded for the *in situ* analysis (see section on technical challenges during deployment).

Sample day	Days deployed	Sample type	***In situ* analysis sampling**			**Archival-M sampling**			**Archival-E sampling**			Comments
			Volume filtered (mL)	Start time	End time	Volume filtered (mL)	Start time	End time	Volume filtered (mL)	Start time	End time	
26-01-18	1	N	1500	08:22	09:16	1500	10:11	11:06				
27-01-18	2	N	1500	08:10	08:59	1500	09:55	10:51				
28-01-18	3	C	0			1500	09:02	09:57				
30-01-18	4	C	0			1500	09:03	09:56				
31-01-18	5	N	1500	08:10	09:03	1500	09:58	10:54				
01-02-18	6	N	1500	08:10	09:02	1500	09:57	10:52				
02-02-18	7	N	1500	08:10	08:59	1500	09:55	10:50				
03-02-18	8	N	1500	08:10	09:00	1500	09:57	10:56				
04-02-18	9	N	1500	08:10	09:03	1500	09:59	11:01				
09-02-18	14	C	0			1500	09:03	10:03				
10-02-18	15	N	1500	08:10	08:59	1500	09:55	10:52				
11-02-18	16	N	1500	08:10	09:01	1500	09:57	10:52				
12-02-18	17	N	1500	08:10	09:05	1500	10:02	11:02				
13-02-18	18	N	1500	08:10	09:08	1500	10:04	11:04				
14-02-18	19	N	1500	08:10	09:11	1500	10:07	11:11				
15-02-18	20	N	1500	08:10	09:14	1500	10:10	11:18				
16-02-18	21	N	1500	08:10	09:08	1500	10:04	11:03				
17-02-18	22	N	1500	08:10	09:08	1500	10:04	11:04				
27-02-18	32	C	0			1500	09:03	10:06				
28-02-18	33	N	1500	08:10	09:11	1500	10:07	11:44				a,c
08-03-18	41	N	674	08:10	08:44	1500[d]	09:41	10:08				a
09-03-18	42	N + E	6	08:10	08:11	1500[d]	09:08	09:36	5	20:05	20:06	b
11-03-18	44	N + E	1500	08:10	09:07	1500	10:04	11:02	1500	20:05	21:04	a,c
12-03-18	45	N + E	1500	08:10	09:09	1500	10:06	11:09	1500	20:05	21:07	
13-03-18	46	N + E	1500	08:10	09:12	1500	10:09	11:09	1500	20:05	21:07	
14-03-18	47	N + E	1500	08:10	09:08	1500	10:05	11:39	1500	20:05	21:04	
15-03-18	48	N + E	1500	08:10	09:14	1500	10:10	11:10	1500	20:05	21:07	
16-03-18	49	N + E	1500	08:10	09:10	1500	10:07	11:06	1500	20:05	21:03	
17-03-18	50	C	0			1500	09:03	10:05				
18-03-18	51	N	255	08:11	08:21	1425	09:18	10:51				

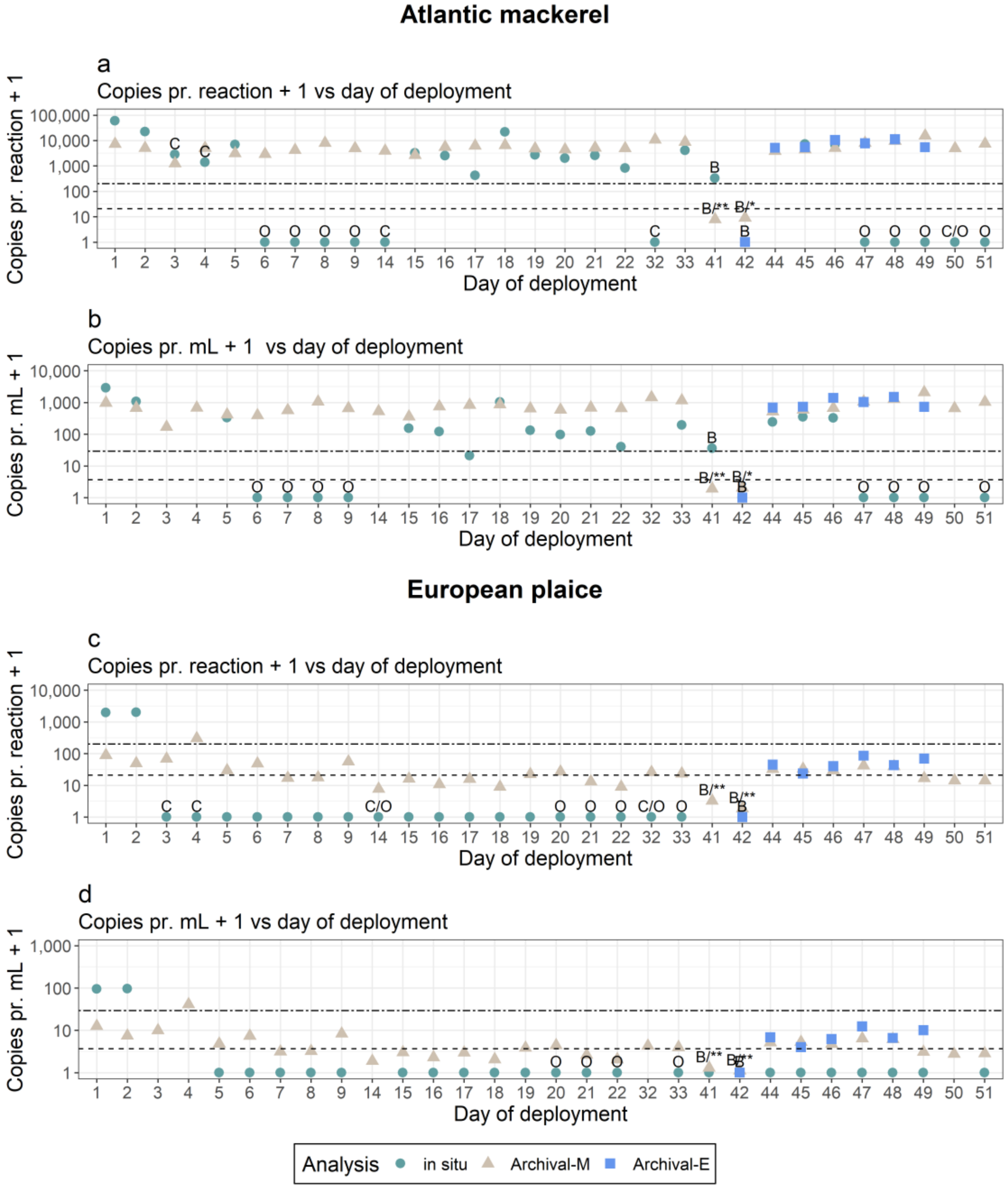

Atlantic mackerel
a
Copies pr. reaction + 1 vs day of deployment
Copies pr. reaction + 1
Day of deployment
b
Copies pr. mL + 1 vs day of deployment
Copies pr. mL + 1
Day of deployment
European plaice
c
Copies pr. reaction + 1 vs day of deployment
Copies pr. reaction + 1
Day of deployment
d
Copies pr. mL + 1 vs day of deployment
Copies pr. mL + 1
Day of deployment
Analysis
in situ
Archival-M
Archival-E

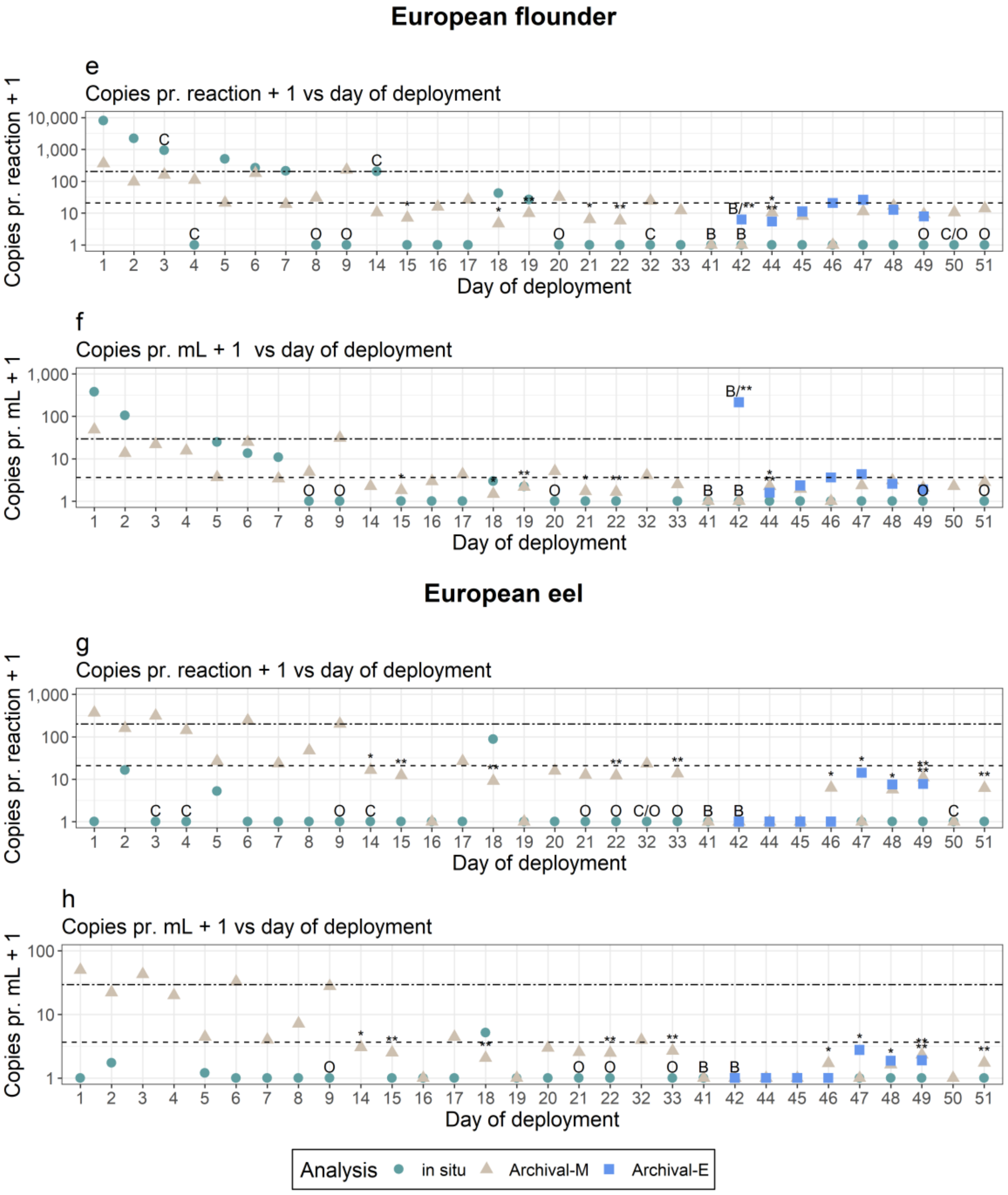

Supplementary figure S1: Time series eDNA results for Atlantic mackerel (a &b), European plaice (c & d), European flounder (e & f) and European eel (g & h).

Results of qPCR analysis for all samples during the deployment in (a, c, e & g) copies/reaction with negative controls, and (b, d, f & h) copies/ml without negative controls. O indicates that an assay was "out of prime" (no reaction). B represents a breakdown of the pump during deployment. C refers to "core negative" control of the entire *in situ* qPCR analysis. (− −) is the LOQ for the laboratory based qPCR analysis and (· −) is the LOQ for the *in situ* analysis. (*) are blank reactions (of three) for each sample.

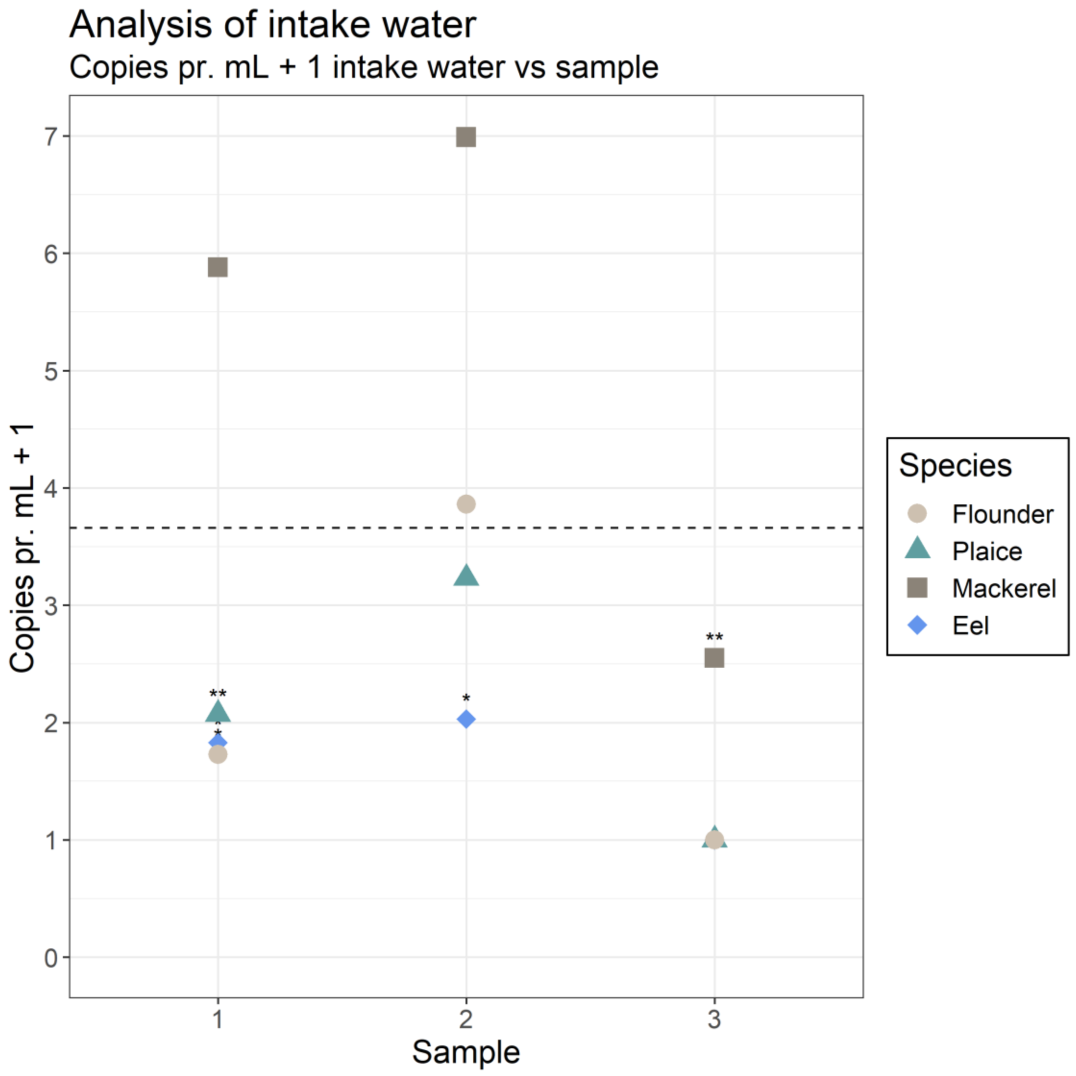

Supplementary figure S2: Analysis of intake water

Analysis of triplicate water samples (1 L) from intake pipe to mesocosm (day 20). (*) show blank reactions (of three) pr. sample. The stippled line (− −) indicates the LOQ. Intake water is daily diluted to 1:10 in the mesocosm (see Methods).

Supplementary table S3: Analysis of exogenous DNA in fish feed

Assay	Feed size (mm)	Potential copies at feeding (cop./mL)[a]	Potential copies at 7h after feeding (cop./mL)[b]		Potential copies at 20h after feeding (cop./mL)[c]		Cop./mg feed
			High decay rate[d]	Low decay rate[e]	High decay rated[d]	Low decay rate[e]	
Mackerel	2	134±14	66±7	91±9	18±2	45±5	6.1E+04
Mackerel	6	67±2	33±1	46±2	9±0	22±1	2.1E+06
Flounder	2	40±8	20±4	27±5	5±1	13±3	1.8E+04
Flounder	6	6[f]	0	0	0	0	2.9E+03
Plaice	2	25±15	12±7	17±10	3±2	8±5	1.1E+04
Plaice	6	0.19±0.14[g]	0	0	0	0	5.9E+03

[a]Potential copies at time of feeding (copies/mL)

[b]Potential copies at 7 h after feeding (copies/mL)

[c]Potential copies at 20 h after feeding (copies/mL)

[d]High decay rate ($\beta = 0.101$)

[e]Low decay rate ($\beta = 0.055$)

[f]Only one reaction amplified

[g]2 of 3 reactions amplified

Model based estimation of potential exogenous DNA concentration in water from fish feed: a) immediately after feeding, b) 7 hours after feeding, corresponding to time between feeding and Archival-E water sampling, and c) 20 hours after feeding, corresponding to time between feeding and start of sampling of the *in situ* analysis and Archival-E samples. All qPCR analyses for European eel were negative. The model based estimations use the highly conservative assumption that all pellets were dissolved without any consumption and that the DNA is subsequently dispersed homogeneously in the tank.

Supplementary table S4: qPCR chemistries and standard curve parameters

qPCR chemistries and standard curve parameters. Primers and probes are adopted from previous eDNA study on marine fish in the Baltic Seas by Knudsen et al. (2019). The primers specific for mackerel are based on the primers used by Knudsen et al., (2019), but have been modified for more optimal specificity. The probes are equipped with FAM-dye in the 5'-end and Iowa Black F quencher (IBFQ) in the 3'-end, and ZEN quenchers inserted in the middle. The modification of the probe quencher types have been conducted to optimize delta fluorescence. The concentration (nmol) per individual reaction is the optimal concentration inferred for the assay in the final qPCR tube. The number of copies of the target-eDNA fragment reported is in copies per mL filtered seawater. The abbreviations used in the columns are 'LOQ' limit of quantification, 'Eff.' efficiency, 'MFB' microfluidics block. The reference 'Ref' column denotes from which study the primers were adopted.

			**StepOnePlus**		**MFB qPCR module** Pre-deployment		**MFB qPCR module** Post-deployment		**MFB qPCR module** Pre- and post-deployment		
**Species targeted**	**Sequences (5'-3')**	**Conc. (nmol) pr. individual reaction**	**Eff % (Slope) (Int.)**	**LOQ (cp/rxn) (cp/mL)**	**Eff % (Slope) (Int.)**	**LOQ (cp/rxn) (cp/mL)**	**Eff % (Slope) (Int.)**	**LOQ (cp/rxn) (cp/mL)**	**Eff % (Slope) (Int.)**	**LOQ (cp/rxn) (cp/mL)**	**Ref**
**Atlantic mackerel**			100.51	20.00	91.95	600.00	87.45	600.00	89.70	600.00	
Forward primer	TCCCTGCTTGGTCTCTGTTTAG	400	-3.31	2.66	-3.53	28.50	-3.66	28.50	-3.60	28.50	Modified from
Reverse primer	GGCGACTGAGTTGAATGCTG	400	42.03		44.22		44.65			44.44	Knudsen *et al.*
Probe	FAM-TTCCCAAAT/ZEN/CCTCACAGGACTATTC-IBFQ	200									2019
Gblock amplicon	TGAAACTTCGGTTCCCTGCTTGGTCTCTGTTTAGCTTCCCAAATCCTCACAGGACTATTCCTTGCAATGCACTACACGCCCGACGTCGAATCAGCATTCAACTCAGTCGCCCATATTTGCCGGGA										
**European flounder**			97.20	20.00	99.34	600.00	76.02	6000.00	87.68	600.00-6000.00	
Forward primer	TAGGCTTTGCAGTTCTCCTT	1200	-3.39	2.66	-3.34	28.50	-4.07	285.00	-3.71	28.5-285	Knudsen *et al.*
Reverse primer	GCAGGCGTAAAGTTGTCCG	200	41.31		41.73		48.42			45.08	2019
Probe	FAM-CACTGGCTT/ZEN/CGCTCGCCCTATTTTC-IBFQ	300									
Gblock amplicon	CACATACAAAGACCTCTTAGGCTTTGCAGTTCTCCTTACTGCACTGGCTTCGCTCGCCCTATTTTCCCCCAATCTCTTAGGAGACCCGGACAACTTTACGCCTGCAAACCCACTCGTCACGCCAC										
**European plaice**			105.52	20.00	102.20	600.00	88.74	6000.00	95.47	600.00-6000.00	
Forward primer	TAGGCTTCGCAGTCCTCCTC	1200	-3.20	2.66	-3.27	28.50	-3.62	285.00	-3.45	28.5-285	
Reverse primer	TTGCAGGCGTGAAGTTGTCT	200	40.00		41.19		42.91			42.05	Knudsen *et al.*
Probe	FAM-CTAAAAGAT/ZEN/TTGGGGAAAATAGGGCGAGT-IBFQ	400									2019
Gblock amplicon	ACATACAAAGACCTCTTAGGCTTCGCAGTCCTCCTCACTGCACTGGCTTCACTCGCCCTATTTTCCCCAAATCTTTTAGGAGACCCAGACAACTTCACGCCTGCAAACCCGCTCGTCACGCCGCC										
**European eel**			103.17	20.00	94.52	600.00	96.49	6000.00	95.50	600.00-6000.00	
Forward primer	ATCTAGCAACGGACCCCTTA	1200	-3.25	2.66	-3.46	28.50	-3.41	285.00	-3.43	28.5-285	Knudsen *et al.*
Reverse primer	TTGGTTGGTTCTAGCCGCA	1200	42.05		42.92		42.96			42.94	2019
Probe	FAM-ACACCACTA/ZEN/CTAGTTTTATCTTGCT-IBFQ	300									
Gblock amplicon	ACAAATACTTATCTAGCAACGGACCCCTTATCAACACCACTACTAGTTTTATCTTGCTGACTTCTACCATTAATAATTTTAGCGAGCCAAAACCACATGCGGCTAGAACCAACCAACCGCCAGCG										

Supplementary table S5: *in vitro* specificity testing

**Species targeted assay**	**Atlantic mackerel**	**European eel**	**European flounder**	**European plaice**
**Positive control**	Atlantic mackerel *(Scromber Scrombus)*	European eel *(Anguilla Anguilla)*	European flounder *(Platichthys flesus)*	European plaice (*Pleuronectes platessa*)
**Non-target species**	Atlantic bonito *(Sarda Sarda)*	European plaice (*Pleuronectes platessa*)	Common dab *(Limanda limanda)*	Common dab *(Limanda limanda)*
**Non-target species**		European flounder *(Platichthys flesus)*	American plaice (*Hippoglossoides platessoides*)	American plaice (*Hippoglossoides platessoides*)
**Non-target species**		Atlantic mackerel *(Scromber Scrombus*	European plaice (*Pleuronectes platessa*)	European flounder *(Platichthys flesus*

For details about the analysis, see Methods section.

Supplementary information on experimental procedures and results

Technical challenges during deployment

During deployment the 12V pump delivering water to the ESP, was replaced because declining pressure was observed. An identical pump was installed for replacement and tested on day 40. However, for unknown reasons the pump efficiency decreased instantly after installation, causing decreasing filtration volume, dropping from 1500 mL to 674 mL on day 41 and to <10 mL on day 42 for the *in situ* analysis. The exact intake volume for the archived samples could not be determined, but was less or equal to the *in situ* analysis, as the filtration of the archival samples was conducted afterward. Due to this, samples taken on day 41 and 42 were excluded from analysis (fig. S1). The "old" pump was put back into operation on day 44 and worked successfully throughout the remainder of the study period.

Laboratory decontamination procedures

Throughout the study we used extensive decontamination procedures, separate laboratories for pre- and post-PCR procedures, and employed rigorous controls to monitor contamination including DNA extraction blanks and triplicate PCR blanks for each qPCR run. For the archival samples, plaice and flounder contamination was observed in the laboratory based extraction negative for archival samples taken on day 1, 2, 3, 4, 5, 6, 14, 15, 16 and 17. Specifically, only 1 of 3 reactions amplified in the PCR blank (Plaice = 36.05 Cycle threshold (Ct) and flounder =38.23 Ct). In both instances, the Ct values were higher (DNA concentration lower) than for any of the Ct values obtained for qPCRs of actual archival samples (max Ct 34.86 and 37.30 for plaice and flounder, respectively). Hence, the contamination observed likely has little to no effect on our results. All other laboratory controls conducted showed no contamination.

In situ analysis reagent assessment

Pre-deployment *in situ* qPCR analysis efficiency was similar to tests performed in the laboratory on the StepOnePlus platform. Post-deployment, flounder and plaice assays decreased in efficiency and for flounder, plaice and eel assay sensitivities were lower, while the mackerel assay was unaffected (Table S4). This difference is likely due to the "onboard" ESP storage at room temperature compared to standard laboratory freezer storage, which can lead to partial destabilization of the assay, thus lowering functionality and sensitivity[1,3]. Assays targeting plaice, flounder and eel were stored onboard the ESP for ~8 months and mackerel for ~5 months prior to deployment. For future deployments, long-term stability tests of reagents are critical as well as minimizing onboard storage before deployment.

In situ internal positive control

In situ analysis of IPC showed an average Ct value of 30.6 ± 0.38 (95% confidence interval, N=6) when amplified with DNase free water. IPC reactions with DNA extracted from the Oceanarium tank water samples were categorized as inhibited when the Ct value was one above the average Ct for the IPC amplified with DNase free water. While the vast majority of samples did not show signs of inhibition, slight inhibition was observed on day 9 (Ct =33.10) and 47 (Ct =32.24). Among all samples from the Oceanarium tank average Ct value was 30.7 ± 0.35 (95% confidence interval, n=25).

References

1. Preston, C. M. *et al.* Underwater application of quantitative PCR on an ocean mooring. *PLoS One* **6**, e22522 (2011).
2. Knudsen, S. W. *et al.* Species-specific detection and quantification of environmental DNA from marine fishes in the Baltic Sea. *J. Exp. Mar. Biol. Ecol.* **510**, 31–45 (2019).
3. Yamahara, K. M. *et al.* Simultaneous monitoring of faecal indicators and harmful algae using an in-situ autonomous sensor. *Lett. Appl. Microbiol.* **61**, 130–138 (2015).
